# Effect of an educational intervention on HPV knowledge and attitudes towards HPV and its vaccines among junior middle school students in Chengdu, China

**DOI:** 10.1186/s12889-019-6823-0

**Published:** 2019-05-02

**Authors:** Chun-Rong Liu, Hao Liang, Xi Zhang, Chen Pu, Qin Li, Qiao-Ling Li, Fei-Yang Ren, Jing Li

**Affiliations:** 1 Chinese Evidence-based Medicine Center and CREAT Group, West China Hospital, Sichuan University , Chengdu, Sichuan 610041 People’s Republic of China; 2 West China School of Public Health and West China Fourth Hospital, Sichuan University, Chengdu, Sichuan 610041 People’s Republic of China; 3 Lung Cancer Center, West China Hospital, Sichuan University, Chengdu, Sichuan 610041 People’s Republic of China; 4 Department of Epidemiology, Cancer Institute of Chinese Academy of Medical Sciences, Peking Union Medical College, Beijing, 100021 People’s Republic of China; 5 Institute of Parasitic Disease, Sichuan Center for Disease Control and Prevention, Chengdu, Sichuan 610041 People’s Republic of China; 6 Department of Hospital Infection Control, Women’s and Children’s Hospital of Sichuan Province, Chengdu, Sichuan 610031 People’s Republic of China; 7 Chengdu Xi-Bei Foreign Language Middle School, Chengdu, Sichuan 610045 People’s Republic of China; 8 Tang Hu Middle School, Chengdu, Sichuan 610200 People’s Republic of China

**Keywords:** Effect, Health education, HPV vaccines, Junior middle school students, China

## Abstract

**Background:**

Little is known about the knowledge and attitudes towards human papillomavirus (HPV) and its vaccines among adolescents in mainland China. Also, limited information has been available on how to improve their knowledge and willingness towards HPV and its vaccines to ensure a successful vaccination program in the future.

**Methods:**

This was a school-based interventional follow-up study. One urban and one rural junior middle school in Chengdu were selected by convenience sampling. At baseline, half of the grade one students were randomly selected as controls and the rest were interventions. A set of self-administered questionnaires on HPV and its vaccines were completed by both groups at baseline. After that, only the intervention group received a PowerPoint-oriented health education and finished the post-education questionnaires. One year later, both groups completed the same questionnaires as the follow-up survey.

**Results:**

In total, 1675 students finished the pre-intervention questionnaires; 751 were from the control group and 924 were from the intervention group. Among them, only 34.3% had heard of cervical cancer/genital warts, while only 15.1% of them had ever heard of HPV. However, 55.2% of students showed their willingness to be vaccinated even before any intervention. Seven variables were found to be associated with the willingness to be vaccinated at baseline. Immediately after the intervention, 88.4% of students were willing to vaccinate themselves. After 1 year, the effectiveness of intervention remained but decreased. Compared with the control group, the intervention group was more aware about cervical cancer, HPV and its vaccines with statistical significance. However, the level of HPV knowledge and willingness to be vaccinated among the intervention group had significantly decreased compared with that immediately after the intervention (*P* < 0.001).

**Conclusions:**

The baseline level of knowledge on HPV, its vaccines, and cervical cancer was very low among junior middle school students in Chengdu, China. However, the willingness to be vaccinated seemed positive. School-based health education is effective and appropriate in increasing the awareness of HPV and willingness towards its vaccines. Regular health education on HPV and cervical cancer prevention at a shorter interval should be guaranteed to ensure continuous effectiveness.

## Background

Cervical cancer is the second most common cancer among females in the developing countries, such as China, due to the lack of effective screening and preventive programs [1, 2]. In mainland China, the incidence of cervical cancer has increased from 6.78/100,000 women in year 2008 to 10.31/100,000 women in year 2013 [3]. The increasing trend was obvious especially in young women [4], which enlarged the overall disease burden in China. Persistent infection with high-risk human papillomavirus (hrHPV), mainly including HPV 16 and 18, has been proven to be the primary cause for cervical cancer [5], and primary prevention based on prophylactic vaccines is key for cervical cancer prevention in the future.

After being first licensed in 2006, prophylactic HPV vaccines have been demonstrated to be effective and safe to prevent the development of high-grade cervical neoplasias [6, 7], particularly when given to girls before sexual onset. Currently, the bivalent vaccine for HPV 16 and 18 (Cervarix®, GlaxoSmithKline Biologicals, Rixensart, Belgium) and the quadrivalent vaccine for HPV 6, 11, 16, and 18 (Gardasil®, Merck and Co., Whitehouse Station, NJ), have been licensed by the China Food and Drug Administration in July 2016 and May 2017, respectively [8, 9], while the nonavalent vaccine has also been conditionally approved by the Chinese National Medical Products Administration through a fast track in April 2018 [10]. Chinese females aged 9~45 years old were recommended for the bivalent vaccine [11], while females aged 20–45 years old for quadrivalent vaccine and 19–26 years old for nonavalent vaccine [10]. The vaccines provide the potentiality to decrease the incidence of cervical cancer after widespread use, especially among the youth before sexual debut.

Adolescents, especially junior middle school students, are the primary candidates for prophylactic HPV vaccination according to the WHO recommendations [12, 13]. However, some previous studies have shown that adolescents had poor knowledge about HPV and its vaccines [14–16], which may pose potential barriers for the promotion of HPV vaccination throughout this country. Education aiming to improve knowledge of HPV and its vaccines among this population has become extraordinarily important as it plays a key role in improving vaccination compliance among adolescents [14]. However, except for few studies conducted in Hong Kong [14], little is known about the knowledge and willingness towards HPV and its vaccines among adolescents in mainland China. Furthermore, limited information was also available on how to improve their knowledge and willingness towards HPV and its vaccines to ensure a successful vaccination program in the future.

Therefore, we conducted a school-based interventional follow-up study aiming to explore the baseline knowledge on HPV and its vaccines among adolescents in Chengdu, a metropolitan city in mainland China, and to evaluate the effectiveness of a health education both immediately after the lecture and at a 1-year interval.

## Methods

### Design and participants

This was a school-based interventional follow-up study from November 2015 to December 2017. This study was conducted in the junior middle schools in Chengdu, which is the provincial capital of Sichuan province in Southwest China. In China, junior middle school students are adolescents aged from 10 to 14 years old (grade one to grade three). In this study, two junior middle schools in Chengdu were selected by using convenience sampling, one was an urban school and the other was a rural school. In year 2015 and 2016, grade one students from the preselected schools were invited to participate in this study. Four out of eight classes in grade one were randomly selected as control group and the rest were the interventional classes in the urban school; in the rural school, five out of ten were randomly selected as control classes and the rest were interventional classes. In total, nine classes were selected as controls and the other nine were selected as intervention group each year in 2015 and 2016.

### Research instrument

Self-completed questionnaires before and immediately after the intervention were administered after a detailed explanation by the researchers. The pre-intervention questionnaire included two parts: part one included socio-demographic characteristics (including the date of birth, gender, ethnicity); part two included the following questions: First, have you ever heard of cervical cancer; Second, have you ever heard of HPV; Third, have you ever heard of prophylactic HPV vaccines; And the fourth, if prophylactic HPV vaccines are available, would you be willing to vaccinate yourself?

The post-intervention questionnaire and the 1-year follow-up questionnaire included the same questions as the following: First, what is the main cause for cervical cancer; Second, what is the route of transmission for HPV; Third, what is the best way to prevent HPV infection; Fourth, what is the best time to vaccinate yourself; And the fifth, if prophylactic HPV vaccines are available, would you be willing to vaccinate yourself?

#### Baseline survey and educational intervention

In November 2015 and December 2016, before the intervention, students in both control and intervention classes completed the pre-intervention questionnaires after a detailed explanation by the researchers. Then all completed questionnaires were collected and checked by the onsite researchers. Then a 1-h interventional PowerPoint (PPT) presentation was given to the interventional groups by the researcher. The PPT included the following eight topics: what is the cervix and what is cervical cancer; Disease burden of cervical cancer in China and worldwide; Who is at risk to develop cervical cancer; What is HPV and the relationship between HPV and cervical cancer; How to prevent HPV infection and cervical cancer; What is an HPV vaccine, how can it prevent cervical cancer, its safety, efficacy, and side effect; Who needs to get vaccinated and how and where to get vaccinated; Questions and answers. After the intervention, all students in the intervention group were asked to complete the post-intervention questionnaires.

#### One-year follow-up

**Fig. 1:**
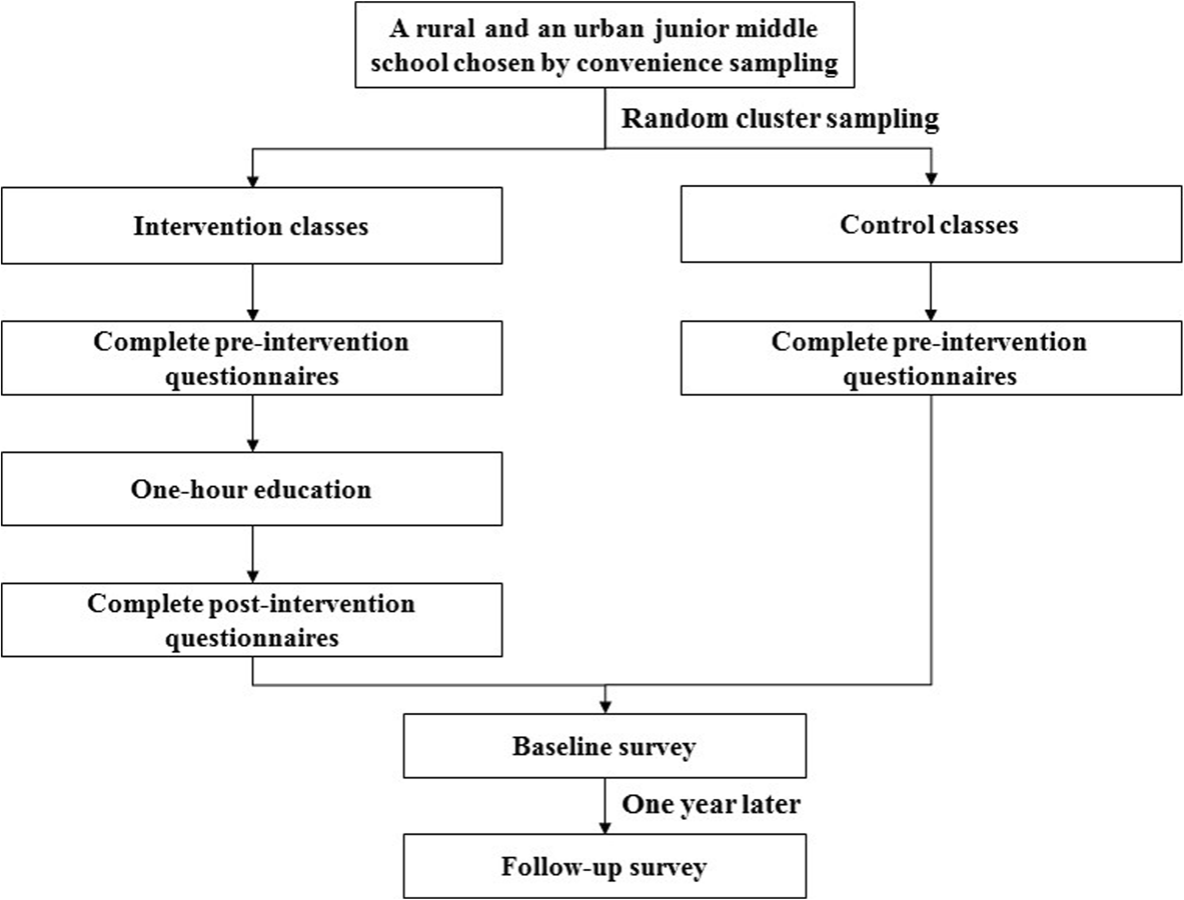
study flowchart. Figure legends: this figure describes this study flowchart

In December 2016 and December 2017, students in both control and intervention classes were organized in their classrooms to complete the 1-year follow-up questionnaires. Again, the questionnaires were explained in details by the researchers. When finished, all completed questionnaires were collected and checked by the onsite researchers and teachers. The study flowchart was shown Fig. 1.

### Data collection and quality control

Two graduate students were assigned to double-enter data from the paper to computer-based database (EpiData 3.1) independently after training. All finished double-entry databases were validated by running EpiData. Any inconsistency found between the two databases was adjusted based on the original paper-based questionnaires until the databases agreed. As final check, one of databases was chosen and underwent a final consistency check. Logic errors (e.g. a student who reported never heard of HPV vaccines knew that HPV vaccines were useful for the prevention from cervical cancer) were again double checked and revised. After the consistency and logic checks, the database was ready for final analysis.

### Statistical analysis

Frequency and percentages were used to describe the characteristics of participants, knowledge on HPV and its vaccines, as well as attitudes towards HPV vaccination. Multivariable logistic regression was used to evaluate the factors associated with the willingness to be vaccinated. Contingency tables using Chi-squared were used to analyze the difference between the control and intervention groups at baseline. They were also used to compare the knowledge between the control and intervention groups at 1-year interval. SPSS statistical software version 18.0 was used to analyze the data. Statistical significance was assessed by two-tailed tests with α level of 0.05.

## Results

### Participants’ profile

Of all, 1675 students have finished the pre-intervention questionnaires, with 753 in year 2015 and 922 in year 2016, respectively. Among them, 751 were in controls and 924 were in the intervention group. Demographic characteristics were presented in Table 1. There were 885 boys (52.8%) and 769 girls (45.9%), with 21 (1.3%) students who did not fill in their gender information; the mean age was 12.31 yrs. (SD = 0.53). The majority (93.6%) of students were Han Chinese. In the intervention group, 99.4% (918/924) of them had finished the post-intervention questionnaires immediately after the health education. After 1 year, 88.5% (1482/1675) of students were followed up, the rate of follow-up was 86.8% (652/751) and 89.8% (830/924) for the control and the intervention group, respectively.

**Table 1:** Demographic characteristic of study participants at baseline

Variables	Number (n)	Percentage (%)
School type		
Rural	838	50.0
Urban	837	50.0
Gender		
Boys	885	52.8
Girls	769	45.9
Missing	21	1.3
Ethnicity		
Han	1579	94.3
Others	49	2.9
Missing	47	2.9
Year		
2015	753	45.0
2016	922	55.0

### HPV awareness and attitudes towards HPV vaccines at baseline

At baseline, only 34.3% (570/1659)of students reported to have ever heard of cervical cancer and/or genital warts. When asked about HPV, only 15.1% (216/1649) of students reported to have heard of it. Also, the low proportion (17.5%) was seen when students were asked if they have ever heard of HPV vaccines, and it was higher in the controls than that in the interventions (22.4% vs. 13.4%, *P<*0.001). However, more than half of them (55.2%) were willing to vaccinate themselves even before our health education (Table 2).

**Table 2:** Awareness and attitudes towards HPV/HPV vaccines among students at baseline

Items	Control	Intervention	Total^a^	*P* ^b^
	n (%)	n (%)	n (%)	
Have you ever heard of cervical cancer/genital warts				
Yes	248 (33.6)	322 (35.0)	570 (34.3)	0.563
No	490 (66.4)	599 (65.0)	1089 (65.7)	
Have you ever heard of HPV				
Yes	106 (14.5)	110 (12.0)	216 (15.1)	0.142
No	627 (85.5)	806 (88.0)	1433 (84.9)	
Have you ever heard of HPV vaccines				
Yes	165 (22.4)	123 (13.4)	288 (17.5)	**<** 0.001
No	571 (77.6)	795 (86.6)	1366 (82.5)	
Are you willing to be vaccinated when HPV vaccines become available?				
Yes	338 (53.3)	503 (56.5)	841 (55.2)	0.215
No	296 (46.7)	387 (42.5)	683 (44.8)	

^a^Numbers do not add up to 1675 due to some incomplete answers

^b^Chi-squared test

### Factors associated with the willingness to be vaccinated

As shown in Table 3, seven variables were found to be associated with the willingness to be vaccinated at baseline with statistical significance by using multivariable logistic regression analysis. Students from the rural school were more likely to receive HPV vaccines than students in the urban school (adjusted odd ratio (OR): 1.51, 95% confidence internal (CI): 1.19–1.91). Compared with data in year 2016, students investigated in year 2015 were more willing to be vaccinated (adjusted OR: 3.93, 95% CI: 3.03–5.09). Students who have ever received sexual health education (adjusted OR: 1.41, 95%CI: 1.09–1.83) or regarded menstruation/spermatorrhoea as a normal physiological phenomenon (adjusted OR: 1.60, 95% CI: 1.13–2.26) showed more positive attitudes toward HPV vaccination. Besides, students who wanted to learn more adolescent sexual health (adjusted OR: 1.66, 95% CI: 1.26–2.17) or concerned more about cervical cancer (adjusted OR: 1.60, 95% CI: 1.13–2.26) would be more willing to be vaccinated. In addition, students who ever heard of HPV vaccines also showed more positive attitudes toward HPV vaccines (adjusted OR: 1.56, 95% CI: 1.14–2.14). Other five factors (age, gender, ethnicity, heard of HPV, heard of cervical cancer) were excluded from this multivariable logistic regression model.

**Table 3:** Factors associated with willingness to be vaccinated among junior middle school students before intervention

Items	Total^a^ (%)	Unwilling to be vaccinated (%)	Willing to be vaccinated (%)	Adjusted OR^b^	95%CI	*P*
School type						
Urban	740 (48.6)	358 (52.4)	382 (45.4)	Ref		
Rural	784 (51.4)	325 (47.6)	459 (54.6)	1.51	1.19–1.91	<0.001
Year						
2016	899 (59.0)	492 (72.0)	407 (48.4)	Ref		
2015	625 (41.0)	191 (28.0)	434 (51.6)	3.93	3.03–5.09	0.001
Received sexual education/knowledge						
No	550 (36.8)	283 (42.4)	267 (32.2)	Ref		
Yes	945 (63.2)	384 (57.6)	561 (67.8)	1.41	1.09–1.83	0.008
Menstruation/spermatorrhea is a normal physiology phenomenon						
No/not sure	254 (16.9)	134 (20.1)	120 (14.4)	Ref		
Yes	1247 (83.1)	533 (79.9)	714 (85.6)	1.60	1.13–2.26	0.008
Willing to get more knowledge of adolescent sexual health						
No	473 (31.3)	261 (38.8)	212 (25.4)	Ref		
Yes	1036 (68.7)	412 (61.2)	624 (74.6)	1.66	1.26–2.17	<0.001
Cervical cancer is relative to me						
No	1026 (68.7)	502 (75.1)	524 (63.5)	Ref		
Yes	467 (31.3)	166 (24.9)	301 (36.5)	1.75	1.35–2.28	<0.001
Heard of HPV vaccines						
No	1232 (81.5)	581 (85.8)	651 (78.0)	Ref		
Yes	280 (18.5)	96 (14.2)	184 (22.0)	1.56	1.14–2.14	0.006

^a^The total number is not added up to 1675 due to some incomplete answers

^b^Adjusted OR, adjusted odds ratio using multivariable logistic regression mode, and five factors (age, gender, race, heard of HPV, heard of cervical cancer) were excluded from this multivariable logistic regression model

### Knowledge and attitudes towards HPV /HPV vaccines immediately after the intervention and at one-year follow-up

Immediately after the 1-h health education, the percentage of willingness to be vaccinated among the intervention group significantly increased by 31.9%, from 56.5 to 88.4% (*P* **<** 0.001). Even, 1 year after the intervention, compared with those in the control group, students in the intervention group were more likely to be aware of HPV and its vaccines regarding the following four items: First, cause for cervical cancer (36.0% vs. 23.1%, *P* **<** 0.001*)*; Second, the transmission route of HPV (63.8% vs. 46.5%, *P* **<** 0.001); Third, the most effective way to prevent HPV infection (71.4% vs. 54.3%, *P* **=** 0.001); And the fourth, age in junior middle school is the best age to be vaccinated (36.9% vs. 17.8%, *P* **<** 0.001) (Table 4). In addition, the willingness to vaccinate themselves was higher in the intervention group than that in the control group (78.8% vs. 68.1%, *P*<0.001) (Table 4).

**Table 4:** Awareness and attitudes between two groups at one-year follow up

Items	Control group	Intervention group	*P* ^b^
	n (%)	n (%)	
Cause for cervical cancer			
HPV infection	150 (23.1)	298 (36.0)	< 0.001
Sexually transmitted disease	101 (15.6)	177 (21.4)	
Other answers^c^	398 (61.3)	353 (42.6)	
Total^a^	649	828	
The transmission route of HPV			
Sexual transmission	289 (46.5)	518 (63.8)	< 0.001
Other answers^d^	332 (53.5)	294 (36.2)	
Total^a^	621	812	
The most effective method to prevent HPV infection			
HPV vaccination	348 (54.3)	586 (71.4)	0.001
Other answers^e^	293 (45.7)	235 (28.6)	
Total^a^	641	821	
The best age to get vaccinated			
Primary school and below	153 (23.7)	165 (20.0)	< 0.001
Junior high school	115 (17.8)	305 (36.9)	
Senior high school and above	125 (19.3)	148 (17.9)	
Anytime/no answer	253 (39.2)	209 (25.3)	
Total^a^	646	827	
Are you willing to vaccinate yourself?			
Yes	438 (68.1)	647 (78.8)	< 0.001
No	205 (31.9)	172 (21.2)	
Total^a^	643	821	

^a^Numbers do not add up to 652 in the control group and to 830 in the intervention group due to some incomplete answers

^b^Chi-squared test

^c^Including: heredity, multiple sexual partners, bacterial infection, bad health habits, smoking and other unhealthy life style, no answer

^d^Including: airborne transmission, food-borne transmission, all of the three routes

^e^Including: regular condom use, sexual onset at early age, avoiding multiple sexual partners, no answer

### Changes of awareness and attitudes among the interventional group between the post-intervention and the follow-up survey

Compared with answers in the post-intervention survey, data from the 1-year follow-up in the intervention group showed decreased level regarding the knowledge on HPV and its vaccines, including the following four aspects: First, cause for cervical cancer (22.3% decrease, from 58.3 to 36.0%, *P* **<** 0.001*)*; Second, the transmission route of HPV (23.1% decrease, from 86.9 to 63.8%, *P* **<** 0.001); Third, the most effective way to prevent HPV infection (14.7% decrease, from 86.1 to 71.4%, *P* **<** 0.001); And the fourth, age in junior middle school is best to get vaccinated (48.0% decrease, from 84.9 to 36.9%, *P* **<** 0.001) (Table 5). The proportion of positive attitudes to vaccinate themselves also decreased from 88.4 to 78.8% (Table 5).

**Table 5:** Changes of awareness and attitudes 1 year after the intervention among the interventional group

Items	Immediately after the intervention	Follow-up after 1 year	Change	*P* ^a^
	n (%)	n (%)	Percentage point difference (%)	
Cause for cervical cancer				
Infected by HPV	534 (58.3)	298 (36.0)	−22.3	< 0.001
Other answers^d^	382 (41.7)	530 (64.0)		
Total	916^b^	828^c^		
The transmitted route of HPV				
Sexual transmission	787 (86.9)	518 (63.8)	−23.1	< 0.001
Other answers^e^	119 (13.1)	294 (36.2)		
Total	906^b^	812^c^		
The most effective method to prevent HPV infection				
HPV vaccination	790 (86.1)	586 (71.4)	−14.7	< 0.001
Other answers^f^	122 (13.3)	235 (28.6)		
Total	912^b^	821^c^		
The best time stage to HPV vaccination s				
Junior middle school	778 (84.9)	305 (36.9)	−48.0	< 0.001
Other answers	138 (15.1)	522 (63.1)		
Total	916^b^	827^c^		
Are you willing to vaccinate yourself?				
Yes	808 (88.4)	647 (78.8)	−9.6	< 0.001
No	106 (11.6)	174 (21.2)		
Total	914^b^	821^c^		

^a^ Chi-squared test

^b^Numbers are not equal to 918 due to some incomplete answers

^c^Numbers are not equal to 830 due to some incomplete answers

^d^including: heredity, sexually transmitted disease, multiple sexual partners, bacterial infection, bad health habits, smoking and other unhealthy lifestyle, no answer

^e^including: airborne transmission, food-borne transmission, all of the three routes

^f^regular condom use, sexual onset at early age, avoiding multiple sexual partners, no answer

## Discussion

This study investigated the baseline knowledge on cervical cancer and HPV among junior high middle school students in Chengdu, China and their willingness towards HPV vaccines. To our knowledge, this is the first study in mainland China that evaluated the effect of a PPT-oriented health education on changing of the awareness and attitudes towards cervical cancer, HPV and its vaccines both immediately after the intervention and at a 1-year interval.

HPV vaccines have been proven effective and safe for prevention of HPV infection [17–19], which had ever decreased the incidence of cervical cytological and histological abnormalities [20]. The recommended primary target population is adolescents of 9–13 years old according to the recent WHO position paper [13]. In mainland China, it was reported that the 15–19-year-olds had the highest infection of hrHPV [21], and the average age of sexual debut was 17 years old [22]. Students in junior middle schools, aged 10–14 years old, would be the best candidates for HPV vaccination in the future. It becomes critical to understand how this population think about cervical cancer, HPV and its vaccines, as well as how to educate this population to ensure a successful vaccination program that aims to decrease cervical cancer and HPV related disease burden in the future.

Our results showed that only 34.3% of students ever heard of cervical cancer at baseline, and it was similar to the findings from another study among the middle school students [23]; however, this proportion was much lower than that among the adult females and the college girls [24, 25]. In our study, before health education, only 15.1% of students had heard of HPV, while 17.5% of them had heard of HPV vaccines, these data were little lower than the results from a similar study in Jinan (15.5% for HPV, 18.9% for HPV vaccines) [23]. Although the low awareness on cervical cancer, the willingness to accept HPV vaccination was high (55.2%), this might because of the common sense that vaccination is effective to prevent diseases.

It was found that students from the urban school were less willing to accept HPV vaccination than those from the rural school, which was consistent with the study among Chinese adult females [26], however, it was opposite to another study finding among adolescents [23]. Students surveyed in year 2016 showed less willingness to be vaccinated than those surveyed in year 2015, the same trend was found in the Jinan study [23]. The possible reason might be that students surveyed in 2016 may be more concerned about the safety and efficiency of the vaccines in the Chinese population because the bivalent vaccine was licensed in mainland China in July 2016.

Similar to another study among Chinese students [27], our study also found that students who received sexual education/knowledge, considered menstruation/spermatorrhea as a normal physiological phenomenon and hoped to learn more adolescent sexual health were more likely to accept HPV vaccination. This finding indicates that correct sexual health knowledge is positively related to the attitudes towards HPV vaccination, and it highly suggests that the HPV related knowledge should be integrated into the routine school-based sexual education to effectively increase the acceptability towards HPV vaccines among adolescents. Our study also found that students who had heard of HPV vaccines and thought HPV was related to their health were more likely to be vaccinated and this was consistent with a previous study [28]. Limited knowledge on the safety and efficacy of HPV vaccines due to the low knowledge level on HPV and its vaccines [29] were shown to be the main concerns from the Chinese population that reduced the acceptance to HPV vaccination [30]. Thus, great efforts such as school-based HPV and cervical cancer related health education should be taken to improve the awareness on HPV and its vaccines especially among the adolescents.

In our study, the proportion of willingness to vaccinate themselves in the intervention group increased by 31.9% immediately after the intervention, which indicated the effectiveness of our health education. Previous studies also showed that school-based lessons were reliable and effective in improving HPV knowledge among students [31] and in increasing their willingness to be vaccinated [14, 32]. Some other studies also found that lecture-based health education had positive influence on improving the knowledge of HPV vaccines and willingness towards HPV vaccination among the rural and employed females in mainland China [25, 33].

After 1 year, the perceived level of knowledge on HPV and the willingness towards HPV vaccination was higher in the intervention group, which indicated the remained effectiveness of the PPT-oriented education. However, the perceived level in the intervention group was obviously lower than that immediately after the intervention due to the lack of a boosting health education during the 1-year interval. Health education on HPV vaccines integrated into a routine sexual education with a shorter interval would be highly recommend in the middle schools to guarantee continuous effectiveness if a high coverage of vaccination would be expected in the future.

Several limitations of this study should be taken into consideration. Firstly, the health education was only a PPT-based oral presentation, and no other types of health education e.g. video based, game-oriented etc. were evaluated in parallel, therefore, we do not know if a health education based on PPT is the best way to increase the knowledge and awareness among adolescents. Secondly, our study was only carried out in two schools of Chengdu and may not be that much representative of the whole city, sampling bias might happen that led to some incomparable variable regarding “have you ever heard of HPV vaccines” between the two groups at baseline. An expanded study would be expected to provide data that are more conclusive. Third, some other factors influencing the acceptability of HPV vaccines were not included in our study, such as the cost of HPV vaccines, parents’ knowledge of HPV vaccines etc. These factors could be very important in deciding whether to be vaccinated or not. If a high acceptance to HPV vaccines would be expected, these factors should also be taken into consideration in the further studies. Last but not the least, there were some incomplete answers for the questions, the researchers should pay more attention to the filed quality control of the future study.

One of the major strengths of this study is that this is the first study that evaluated the effectiveness of a school-based HPV education on changing the awareness and attitudes towards HPV and its vaccines among the junior middle school students immediately and 1 year after the intervention. This study helps to provide important information to the policy makers on how important the health education is, if future decent coverage on HPV vaccination is expected. This study also shows that regular health education is needed to ensure a continuous effectiveness.

## Conclusions

In conclusion, this is the first evaluation of a PPT-oriented health intervention on changing of the awareness and attitudes towards HPV and its vaccines among the junior middle school students in an important city of China. Our results revealed poor HPV related knowledge among junior high school students in Chengdu; however, a comparatively high acceptance towards HPV vaccination was observed even before the education. Our findings also suggest that a school-based education is effective and appropriate in increasing HPV related knowledge and the acceptability towards HPV vaccines among the junior middle school students. Such health education is better to be integrated into the available sexual health education in the middle schools and be provided at a shorter and much regular interval. Since HPV vaccines have been licensed in mainland China in the recent 3 years, our study may be far-reaching to suggest that a longitudinal, nation-wide HPV education campaign suitable for school-based curriculum be advocated.

## Abbreviations

CI: confidence internal
hrHPV: high-risk human papillomavirus
OR: odd ratio
PPT: PowerPoint
